# An integrated analysis based on transcriptome and proteome reveals deastringency-related genes in CPCNA persimmon

**DOI:** 10.1038/srep44671

**Published:** 2017-03-17

**Authors:** Wenxing Chen, Yalou Xiong, Liqing Xu, Qinglin Zhang, Zhengrong Luo

**Affiliations:** 1 Key Laboratory of Horticultural Plant Biology, Huazhong Agricultural University, Wuhan 430070, Hubei, China; 2 Hubei Collaborative Innovation Center for the Characteristic Resources Exploitation of Dabie Mountains, Huanggang Normal University, Huanggang 438000, Hubei, China

## Abstract

Persimmon fruits accumulate a large amount of proanthocyanidins (PAs) during development. PAs cause a dry or puckering sensation due to its astringency. Pollination constant and non-astringent (PCNA) persimmon fruits can lose astringency during fruit ripening. However, little is known about the mechanism of natural de-astringency of Chinese PCNA (CPCNA). To gain insight into the molecular events of CPCNA natural de-astringency, we used mRNA-seq and iTRAQ-based quantitative proteomic analysis to measure changes in genes and proteins expression at two key stages of natural astringency removal (i.e. 10 and 20 weeks after bloom) and water-treated (i.e. 40 °C·12 h) de-astringency fruits. Our analyses show that the three predominantly process in CPCNA de-astringency: (1) water treatment strongly up-regulates glycolysis/acetaldehyde metabolism, (2) expression of genes/proteins involved in PA biosynthetic pathway was remarkably reduced in natural and water-treated de-astringency, (3) sugar metabolism and ethylene related pathway were quite abundant in natural de-astringency. We also found ethylene-related TFs were quite abundant in natural de-astringency, followed by *WRKY* and *NAC* transcription factors. These results provide an initial understanding of the predominantly biological processes underlying the natural de-astringency and “coagulation effect” in CPCNA.

Persimmon (*Diospyros kaki* Thunb.; 2n = 6x = 90) originated in China and has been widely cultivated in Eastern Asia^1^. Most persimmon fruits accumulate large amounts of high-molecular-weight proanthocyanidins (PAs) in special compartment cells called “tannin cells” that cause a strong astringency sensation in fresh fruits; therefore, these fruits are inedible without artificial treatment to remove the astringency^2^. There is a spontaneous mutant phenotype whose fruits naturally lose their astringency on the tree^1^, these fruits are edible without any artificial treatment after harvest. This non-astringent mutant phenotype, also called pollination constant and non-astringent (PCNA), includes Japanese PCNA (JPCNA) and Chinese PCNA (CPCNA). The genetic difference of the natural de-astringency trait between CPCNA and JPCNA is a single dominant locus controlling in the former genotype but recessive in the latter one^3^,^4^.

PAs, also called condensed tannins, are plant secondary metabolites that are synthesized via the shikimate and flavonoid pathway^5^,^6^. The genetics and biochemistry of the flavonoid pathway have been characterized in several plant species^7^,^8^,^9^. Most of the enzymatic structural genes involved in PA biosynthesis, such as phenylalanine ammonia-lyase (*PAL*), chalcone synthase (*CHS*), chalcone isomerase (*CHI*), flavanone-3-hydroxylase (*F3H*), flavonoid 3′5′-hydroxylase (*F3*′*5*′*H*), dihydroflavonol 4 reductase (*DFR*), anthocyanidin synthase (*ANS*), leucoanthocyanidin reductase (*LAR*), and anthocyanidin reductase (*ANR*), have been isolated^10^,^11^,^12^. Other genes involved in PA accumulation have been identified in *Arabidopsis*, such as *TT12* encoding a multidrug and toxic compound extrusion family transporter (MATE)^13^, *TT19* encoding glutathione S-transferase (GST)^14^, and autoinhibited H^+^-ATPase isoform 10, which is required to transport PAs to the vacuole^15^. *DkLAC1*, a *Laccase* gene isolated from CPCNA, is phylogenetically related to TT10/AtLAC15, which is potentially involved in the polymerization of PAs monomers in persimmon fruit^16^.

Soluble tannins cause astringency in persimmon^2^. Acetaldehyde was found to accumulate significantly in the flesh following the treatment of fruits with warm water^17^, ethanol^18^ or CO_2_^19^,^20^,^21^, and acetaldehyde reacts with soluble tannins to form an insoluble gel, causing the fruits to lose their astringency^22^,^23^,^24^. In addition, astringency removal in soft persimmon is mainly caused by the accumulation of water-soluble pectin^25^. In plants, acetaldehyde can be synthesized from pyruvate and ethanol, catalyzed by Pyruvate decarboxylase (PDC) and Alcohol dehydrogenase (ADH), respectively^26^,^27^,^28^. Recently, eight *DkADH* and *DkPDC* genes were isolated from persimmon fruits, and *DkADH1 and DkPDC2,* have been suggested to be key genes involved in persimmon astringency removal^29^,^30^. And six *DkPK* genes were isolated, which is a key enzyme that catalyzes pyruvate synthesis from phosphoenolpyruvate, and suggested that *DkPK1* plays a potential role involved in CPCNA natural de-astringency^31^. In addition, several hypoxia-responsive *ERF* transcriptional regulator genes were also isolated and characterized^29^,^32^,^33^, in which *DKERF9, DkERF10, DkERF19*, and *DkERF22* activated the promoters of *DkPDC2, DkADH1*, and *DkPDC3* under high CO_2_ (95%) treated de-astringency^29^,^33^. A previous study suggested that the “dilution effect” resulted in natural astringency removal in JPCNA^34^, but this effect was not enough to cause CPCNA fruits to lose astringency; there may be a “coagulation effect” in which soluble tannins converted to insoluble during late stage of CPCNA fruit development^30^,^31^, but the molecular event underlying CPCNA fruits de-astringency was not so clear.

Recently, a genome-wide transcriptome analysis of ‘Luotian-tianshi’ persimmon, a CPCNA fruit treated with ethanol to eliminate its astringency at 10 weeks after bloom (WAB), provided important insight into the process of de-astringency^35^. However, the molecular events and primary course of metabolism in natural de-astringency and the relationship between natural and artificial de-astringency are largely unknown. In this study, fruits at the key stages of natural astringency removal (i.e. 10 and 20 WAB) and water-treated no-astringent fruits were used for RNA-seq and iTRAQ-based proteomic analysis. These transcriptomic and proteomic data were then used to investigate the genes/proteins expression patterns in two different de-astringency processes and to identify statistically robust GO categories and the underlying genes associated with natural astringency removal.

## Results

### PA accumulation patterns between CPCNA, JPCNA and non-PCNA types

**Figure 1:**
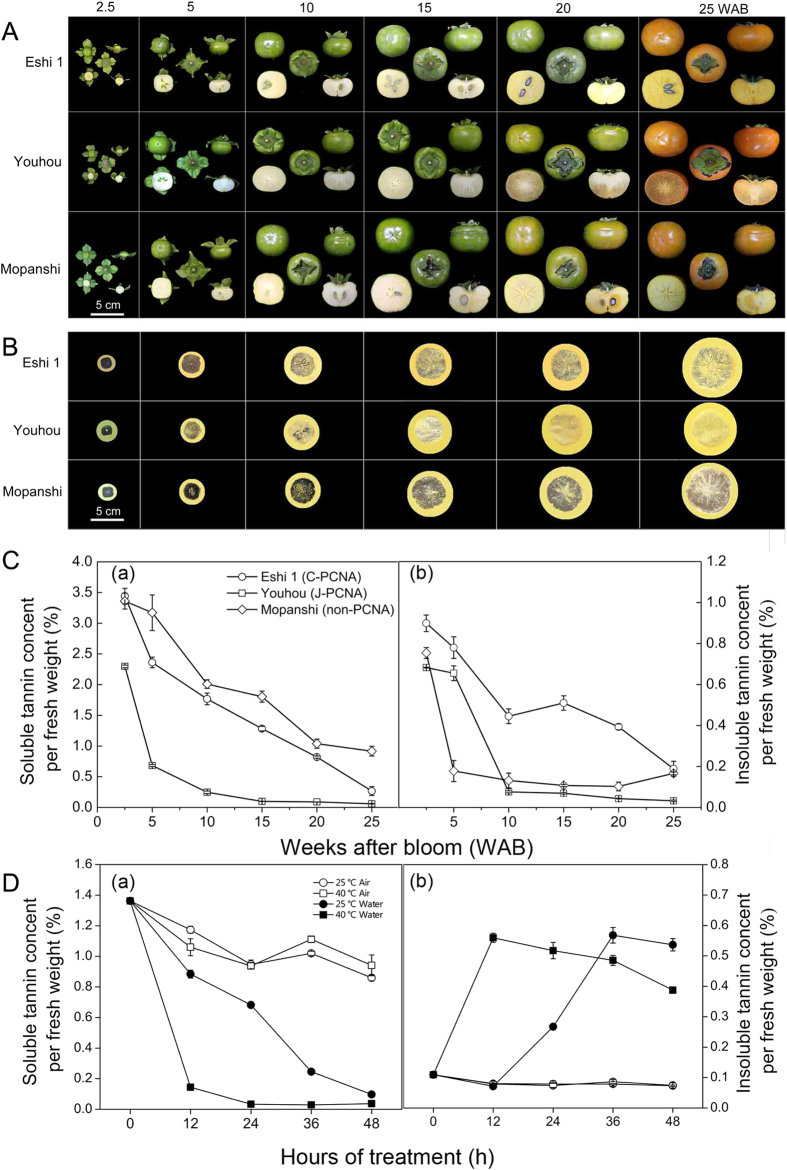
Changes in soluble and insoluble tannin content at different developmental stages and treatments in different persimmon fruits. (**A**) Fruit development pattern at 2.5, 5, 10, 15, 20, and 25 weeks after bloom (WAB). (**B**) Test of the soluble tannin content using the imprint method. FeCl_2_ reacts with soluble tannins, and the darker the resulting product is, the more astringent the fruits are. (**C**) Analysis of the soluble tannin content by the Folin-Ciocalteu method. (a) Soluble tannin content per fresh weight. (b) Insoluble tannin content per fresh weight. Data collection started 2.5 WAB and continued until 25 WAB. (**D**) Measuring the tannin content after water treatment for each time point (40 and 25 °C water treated compared to untreated samples for each time point) of persimmon fruits. (a) Soluble tannin content per fresh weight. (b) Insoluble tannin content per fresh weight. The error bars indicate _SD_. Bar = 5 cm. Three independent group fruits were used for each individual astringency removal type.

To examine the PA accumulation patterns in CPCNA fruits, three cultivars, ‘Eshi 1′ (CPCNA), ‘Youhou’ (JPCNA) and ‘Mopanshi’ (non-PCNA), were used for PA seasonal change determination (Fig. 1A). The imprinting method was performed to approximately measure the soluble tannin content in persimmon fruits. It was deeply stained at 2.5 WAB and gradually lightened with fruit development (Fig. 1B). The stain was lightly at 10 WAB in ‘Youhou’ and later in ‘Eshi 1’ (i.e. 20 WAB), while the ‘Mopanshi’ was darkly stained even at 25 WAB (Fig. 1B).

To confirm the imprinting results, the soluble and insoluble tannin contents were quantified by the Folin-Ciocalteu method^36^. The same trend of soluble tannin concentration change was shown using two measurements (Fig. 1B,C). Previous reports indicated that the soluble tannin concentration is reduced significantly in PCNA type compared to that in non-PCNA during the early stage of fruit development^10^,^12^. This same tendency was confirmed in JPCNA (Fig. 1C); the soluble tannin concentration was obviously reduced after 2.5 WAB. The soluble tannin concentration of JPCNA ‘Youhou’ decreased to 0.2% at 10 WAB, indicating that the fruits at this point had already lost their astringency^2^. Meanwhile, the soluble tannin concentration was maintained at a high level at the same stage in CPCNA and non-PCNA. In addition, the insoluble tannin concentration showed a slight increase in CPCNA after 10 WAB, but no significant change was observed in JPCNA and non-PCNA (Fig. 1C).

### The insoluble tanning content sharply increased while soluble tannin decreased after water treatment

To examine the tannin variation and gene expression changes during artificial de-astringency, the ‘Eshi 1′ (CPCNA) fruits sampled at 10 WAB were then treated with water (25 and 40 °C) to remove their astringency. We found that both 25 and 40 °C water can remove fruits astringency, but the fruits undergoing 25 and 40 °C air cannot eliminate their astringency (Fig. 1D). The soluble tannin was sharply decreased after 12 h treatment, company with insoluble tannin increased of fruits treated with 40 °C water, after 48 h treatment fruits undergoing 25 °C water also lost their astringency (Fig. 1D). This results confirmed that the soluble tannin was insolubilized after water treatment, heat treatment just accelerate fruits astringency removal but not the main factor for fruits astringency lose.

### *De novo* assembly and annotation of CPCNA persimmon transcriptome

To uncover the biological processes underlying the natural and water-treated de-astringency in CPCNA persimmon fruits, we performed whole transcriptome shotgun sequencing over development stages for CPCNA and water treatment. We chose to sample at 10 and 20 WAB in order to capture most of the transcriptional changes, based on the significant change in the PA concentration in CPCNA compared to that in JPCNA and non-PCNA from 10 to 20 WAB. To investigate the differentially expressed genes underlying water treatment, the fruits treated after 12 h with 40 °C water and 25 °C air were also selected for RNA-seq. The correlation matrix shows that the three replicates for each treatment cluster together (Supplementary Fig. S1). The 12 samples produced about 352.8 million paired-end reads, averaging 29.4 million reads per sample (Supplementary Table S1). Filtered and trimmed reads are available via the NCBI Short Read Archive. The assembled transcriptome were annotated with NCBI protein database (Supplementary Table S2) and a total of 84,429 coding sequences (CDs) were predicted, of which 81,317 could be mapped to the protein databases.

As part of Blast2GO, the taxonomic distribution of the transcripts was provided using BLASTX against the non-redundant database (Supplementary Fig. S2A). More than 47% of the unigenes showed top hits to sequences from *Vitis vinifera.* This is consistent with the fact that the grape fruits accumulate a large amount of PAs^37^. Only a few top hits were from *Camelia sinensis*, which also accumulates various polyphenolic compounds^38^.

Among the 86,784 unigenes with at least one GO term assigned, 73,178 unigenes (53.8% of All-unigenes), 54,473 unigenes (40.1% of All-unigenes), and 18,500 unigenes (13.6% of All-unigenes) were assigned to the biological process (GO:0008150), cellular component (GO:0005575), and molecular function (GO:0003674) categories, respectively (Supplementary Dataset S1). The overall distribution and multilevel GO distribution within these broad GO categories are shown in Supplementary Fig. S2B. In addition, 1,908 unigenes were annotated using the Carbohydrate-Active EnZYme Database (http://www.cazy.org/Welcome-to-the-Carbohydrate-Active.html), with the Glycosyl Transferase family being the most abundant class of enzymes, followed by the Glycoside Hydrolase and Carbohydrate Esterase families (Supplementary Fig. S3).

### Differentially expressed genes (DEGs) in CPCNA persimmon fruits undergoing de-astringency naturally or with water treatment

**Figure 2:**
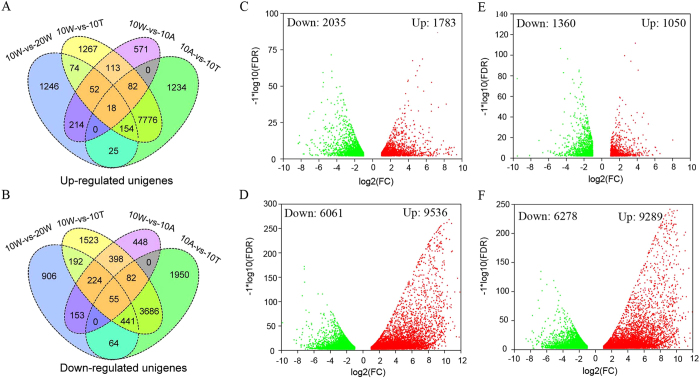
Overview of the differentially expressed genes in natural and water-treated de-astringency (with a FDR value of *P* < 0.05 and an expression level change of at least 2-fold). Venn diagram showing the number of up-regulated (**A**) and down-regulated (**B**) genes that in natural and water-treated de-astringency, respectively. And their relation between the FDR and FC for differentially expressed are shown by volcano plots.10 W-vs-20 W (**C**), 10 W-vs-10 T (**D**), 10 W-vs-10 A (**E**), and 10 A-vs-10 T (**F**), respectively. 10 W: fruits sampled at 10 weeks after bloom (WAB); 20 W: fruits sampled at 20 WAB; 10 T: fruits sampled at 10 WAB and then treated with 40 °C water for 12 h; 10 A: fruits sampled at 10 WAB and then treated with 25 °C air for 12 h.

To identify de-astringency-related genes, we performed a differential gene expression analysis comparing the fruits at 20 WAB (20 W) to fruits at 10 WAB (10 W). A total of 3,818 unigenes were differentially expressed in natural de-astringency, the relation between the FDR (false discovery rate) and FC (fold change) for all DEGs is shown in the volcano plots (Fig. 2C). Supplementary Dataset S2 lists the differentially expressed unigenes with their log2FC, FDR, and their annotation.

To investigate genes response to water treatment, we performed a differential gene expression analysis comparing the fruits treated with 40 °C water at 12 h (10 T) versus the untreated. There are 15,597 unigenes differentially expressed, the relation between the FDR and FC for all DEGs is shown in the volcano plots (Fig. 2D and Supplementary Dataset S3).

To quantify background gene expression changes in the persimmon transcriptome, the 25 °C air-treated fruits at 12 h (10 A) were also selected for gene profiling. We compared the 10 W vs 10 A (10 W-vs-10 A) and 10 A vs 10 T (10A-vs-10 T) (Fig. 2E,F and Supplementary Dataset S4,5), in order to minimize genes that related to de-astringency. The up- and down-regulated genes in each pairwise comparison are exhibited in Venn diagram (Fig. 2A,B). There are a substantial number of transcripts whose expression changed course of the experiment. It is not unexpected some differentially expressed genes most likely results from a combination of response to the abiology stress (i.e. the heat stress) and normal circadian expression cycles. By incorporating control sample (10 A), we were able to identify deastringency-related genes against this background of fluctuating gene expression. However, it is likely that the expression level of some genes reflects both the effect of water treatment and changes in background expression.

### KEGG pathway and Gene Ontology enrichment analysis suggests de-astringency naturally or with water treatment related pathway

**Figure 3:**
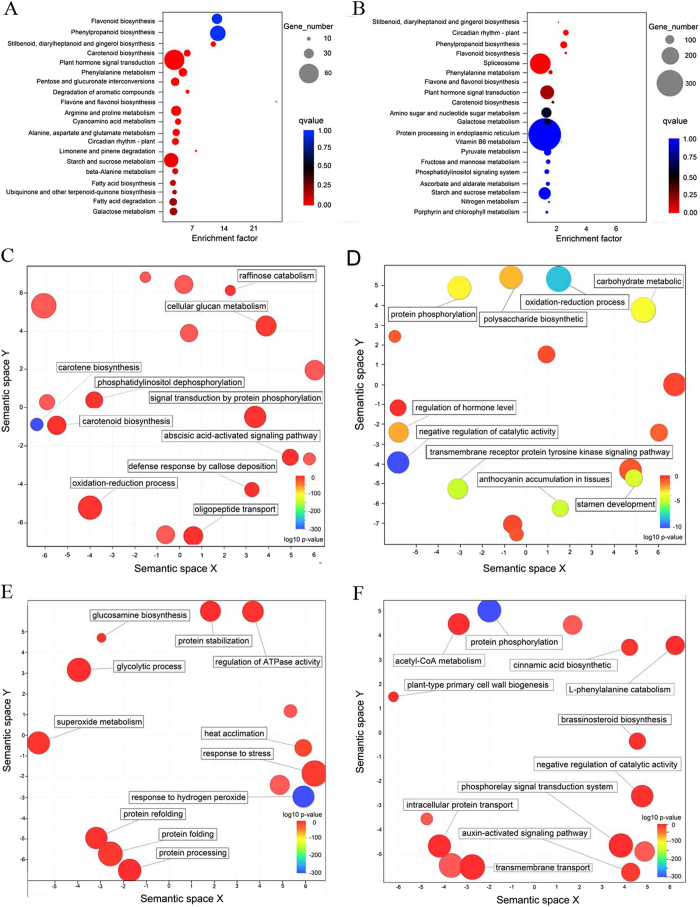
GO and KEGG enrichment analyses of DEGs in natural and water-treated de-astringency. Scatterplot of enriched KEGG pathways for DEGs in natural de-astringency (10 W-vs-20 W) (**A**) and in water-treated de-astringency (10 W-vs-10 T) (**B**). The enrichment factor indicates the ratio of the differentially expressed gene number to the total gene number in a certain pathway. The size and color of the dots represent the gene number and the range of *P* values, respectively. GO enrichment analysis of biological processes in 10 W-vs-20 W and in10 W-vs-10 T on a subset of genes that are up-regulated (**C** and **E**) and a subset of genes that are down-regulated (**D** and **F**), respectively. To identify similar GO terms among the enriched terms, this set of GO terms was categorized using semantic clustering (REVIGO). Each ball represents a cluster of GO terms related to a similar process, and the size of the ball represents the number of GO terms grouped in that cluster. The color of the balls indicates the *P* value of the GO enrichment analysis; red indicates the highest *P* value and blue the lowest (least likely to occur by chance). The cutoff *P* value for the GO enrichment analysis was set to 0.01. The background used for the GO enrichment analysis was all the annotated unigenes of the assembly.

Scatterplot of enriched KEGG pathways for 3,818 differentially expressed genes of fruits at 20 WAB (10 W-vs-20 W) found PA biosynthesis related pathway (i.e. flavonoid biosynthesis, phenylpropanoid biosynthesis, and flavone and flavonol biosynthesis), sugar metabolism (i.e. pentose and glucuronate interconversions, starch and sucrose metabolism, and galactose metabolism), and fruit color related pathway (i.e. carotenoid biosynthesis) (Fig. 3A). The differentially expressed genes were divided into two subsets, one containing genes that up-regulated and the other containing down-regulated genes, and both subjected to Gene Ontology (GO) enrichment analysis and semantic clustering. Among the significantly enriched GO terms found for the set of up-regulated genes, terms related to sugar metabolism (i.e. raffinose catabolic process and cellular glucan metabolic process), fruit color related pathway (carotene biosynthetic process and carotenoid biosynthetic process), signal transduction pathway (signal transduction by phosphorylation, abscisic acid-activated signaling pathway, and ethylene-activated signaling pathway) (Fig. 3C and Supplementary Dataset S6). For the set of down-regulated genes could be associated with a range of process, including negative regulation of catalytic activity, regulation of hormone levels, and anthocyanin accumulation (Fig. 3D and Supplementary Dataset S7). Scatterplot of enriched KEGG pathways for 15,597 differentially expressed genes of undergoing water-treated fruits at 12 h posttreatment (10W-vs-10T) also concerned PA biosynthesis related pathway (i.e. phenylpropanoid biosynthese, flavone and flavonol biosythesis), sugar metabolism (i.e. galactose, fructose, and manose metabolism) and pyruvate metabolism (Fig. 3B). The GO enriched terms of up-regulated by water-treated genes were related to primary glucosamine biosynthesis and glycolytic process (Fig. 3E and Supplementary Dataset S8). And the down-regulated genes were associated with a range of process, including acetyl-CoA metabolic process, cinnamic acid biosynthetic process, plant-type primary cell wall biogenesis, and auxin-activated signaling pathway (Fig. 3F and Supplementary Dataset S9).

### GO enrichment analysis up- and down-regulated genes shared in natural and water-treated de-astringency

**Figure 4:**
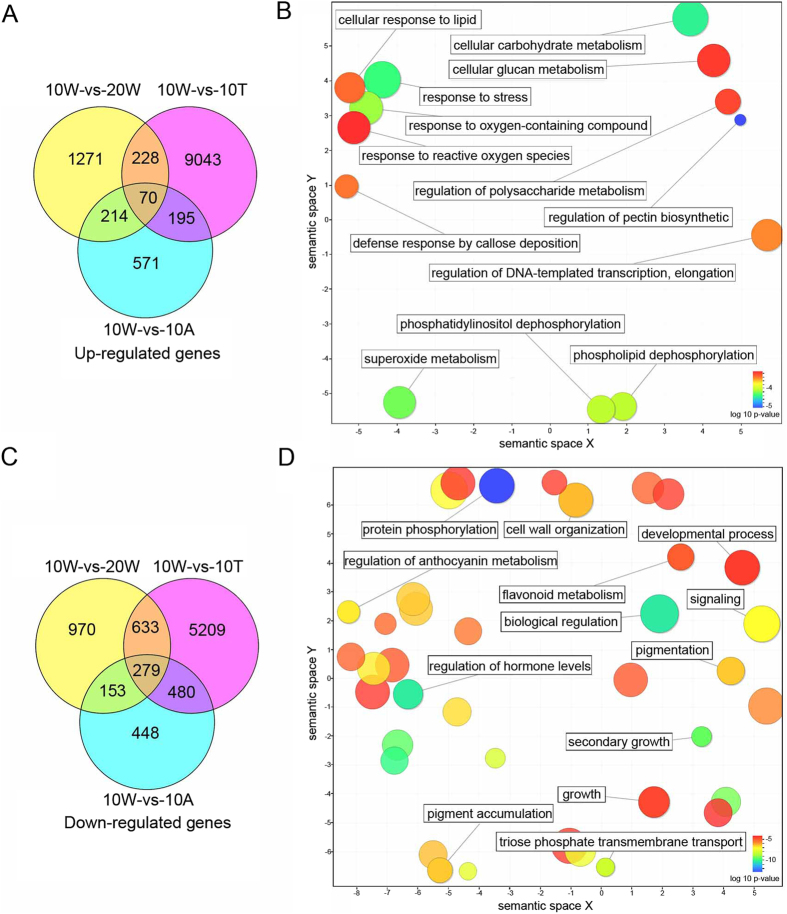
GO enrichment analysis the shared up- and down-regulated genes in natural and water-treated de-astringency. Venn diagram showing the number of up-regulated (**A**) and down-regulated (**C**) genes shared in natural and water-treated de-astringency. (**B**) Top 10 enriched GO terms of the 228 shared up-regulated genes. (**D**) Top 10 enriched GO terms of the 633 shared down-regulated gene. 10 W: fruits sampled at 10 weeks after bloom (WAB); 20 W: fruits sampled at 20 WAB; 10 T: fruits sampled at 10 WAB and then treated with 40 °C water for 12 h; 10 A: fruits sampled at 10 WAB and then treated with 25 °C air for 12 h.

To examine the function of common up- and down-regulated genes in natural and water-treated de-astringency, a GO enrichment analysis was performed. The 228 shared up-regulated genes (Fig. 4A) were significantly enriched in the GO terms that were abundant are related to primary sugar metabolism and stress response (i.e. oxygen response and cellular response to lipid) (Fig. 4B and Supplementary Dataset S10). The 633 shared down-regulated genes (Fig. 4C) in two de-astringency process, the significantly enriched GO terms that were abundant are related to the fruit development (i.e. development process, second growth), pigmentation (i.e. anthocyanin metabolism), and flavonoid metabolism (Fig. 4D and Supplementary Dataset S11).

### Water treatment strongly up-regulates glycolysis/acetaldehyde metabolism in persimmon fruits

**Figure 5:**
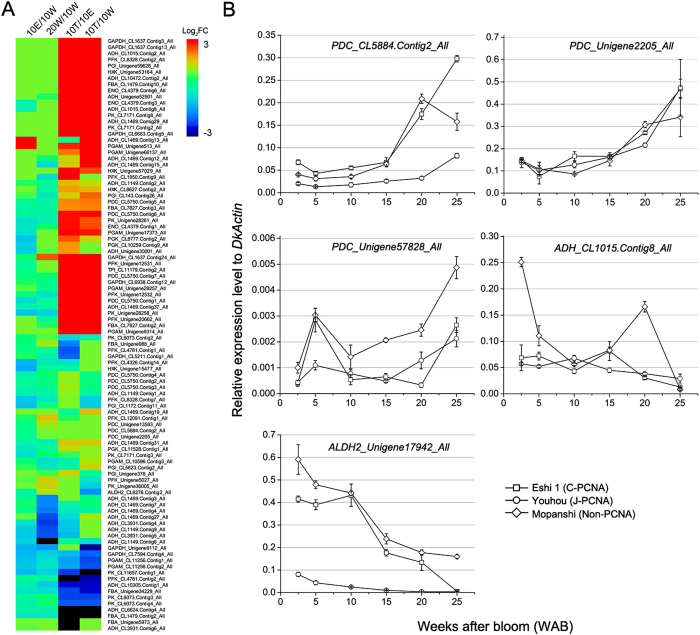
Acetaldehyde metabolism-related genes expression and qRT-PCR validation. (**A**) The expression level of acetaldehyde metabolism-related genes in 10 W-vs-20 W, 10 W-vs-10 T, 10 W-vs-10 A, and 10 A-vs-10 T. The intensity of the Log2 fold change (−3 to 3) is indicated using rainbow scheme; red, up-regulated; blue, down-regulated. (**B**) qRT-PCR analysis to determine the 5 acetaldehyde metabolism-related genes from 2.5 to 25 WAB in the three cultivars. The expression level is shown as the value relative to the expression of *DkActin* (accession no. AB473616). The error bars indicate _SD._ 10 W: fruits sampled at 10 weeks after bloom (WAB); 20 W: fruits sampled at 20 WAB; 10 T: fruits sampled at 10 WAB and then treated with 40 °C water for 12 h; 10 A: fruits sampled at 10 WAB and then treated with 25 °C air for 12 h. FC: the fold change in gene expression.

Both the KEGG pathways and GO enrichment results suggested that glycolysis process and pyruvate biosynthesis is significantly enriched in underlying water-treated fruits (Fig. 3B,E and Supplementary Dataset S8). By mapping genes to pyruvate metabolism pathways, a total of 268 unigenes were found (Table 1), of which 69 differentially expressed, and 57 of them up-regulated, only 12 were down-regulated. The ten step genes for glycolysis process (i.e. *HXK, PGI, PFK, ALD, TPI, GAPDH, PGK, PGAM, NSE*, and *PK*), and genes specific for acetaldehyde biosynthetic (i.e. 14 *PDC-like* and 7 *ADH-like* genes) were strongly up-regulated (i.e. 2- to 1,024-fold) (Fig. 5A). The differentially expressed genes involved in natural de-astringency were also enriched in terms of sugar metabolism (Fig. 3A,C and Supplementary Dataset S6), so we also compared the pyruvate metabolism related genes expression in natural de-astringency process. Several genes (i.e. 2 *PFK*, 1 *ALD*, 1 *GAPDH*, 1 *PK*, and 3 *PDC*) that involved in glycolysis/acetaldehyde were also up-regulated in natural astringency removal (Table 1). This results suggested both in natural and water-treated de-astringency the acetaldehyde metabolism was up-regulated but more remarkable in water treatment.

### Expression of genes involved in PA biosynthetic pathway was highly reduced in natural and water-treated de-astringency

**Figure 6:**
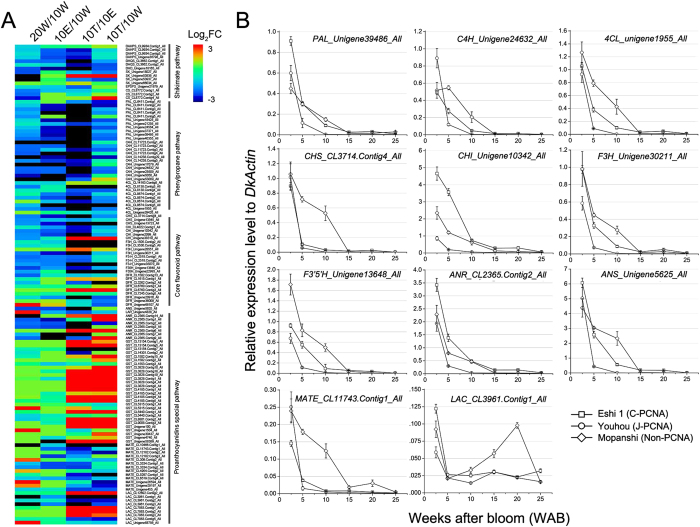
PA biosynthesis-related genes expression and qRT-PCR validation. (**A**) The expression level of PA biosynthesis-related genes in 10 W-vs-20 W, 10 W-vs-10 T, 10 W-vs-10 A, and 10A-vs-10T. The intensity of the Log2 fold change (−3 to 3) is indicated using rainbow scheme; red, up-regulated; blue, down-regulated. (**B**) qRT-PCR analysis to determine the 11 key gene involved in PA biosynthesis. Flesh from three individuals of each cultivar from 2.5 to 25 WAB was used for the expression analysis. The expression level is shown relative to the expression of *DkActin* (accession no. AB473616). Error bars indicate _SD._ 10 W: fruits sampled at 10 weeks after bloom (WAB); 20 W: fruits sampled at 20 WAB; 10 T: fruits sampled at 10 WAB and then treated with 40 °C water for 12 h; 10 A: fruits sampled at 10 WAB and then treated with 25 °C air for 12 h. FC: the fold change in gene expression.

Many of GO terms of down-regulated genes in natural and water-treated de-astringency were related to the flavonoid biosynthesis (Fig. 3D,F and Supplementary Dataset S7,9). Expression of four steps of PA biosynthetic pathway (i.e. shikimate pathway, phenylpropane pathway, core flavonoid pathway, and proanthocyanidins special pathway) genes indicated most of them were down-regulated (Table 2 and Fig. 6A). In the shikimate biosynthetic genes, namely, *DHQS, DHD, SK, CS* was reduced (i.e. 3- to 11-fold) in natural de-astringency, while two *SK-like* and one *CS-like* unigenes increased after water treatment. The genes expression of early flavonoid biosynthetic pathway, including, *C4H, CHS, CHI, F3H, F3*′*H*, and *F3*′*5*′*H* was considerably reduced (i.e. 2- to 14-fold). And some late flavonoid biosynthetic genes were also down-regulated in both natural and water-treated de-astringency. These belong to both the flavonol biosynthetic branch and the proanthocyanidin biosynthetic branch. However, *Flavonol synthase (FLS-like*) gene that specific for flavonol biosynthetic was not differentially expressed in natural de-astringecy (Supplementary Dataset 2). The specific for proanthocyanidin biosynthetic pathway genes, namely, *DFR, ANS, ANR, MATE*, and *LAC* were highly reduced (i.e. 2- to 45-fold). This results suggested that PA biosynthetic pathway genes were specific down-regulated during natural de-astringency.

Next, we performed a targeted analysis of PA biosynthesis pathway-related genes, that were significantly down-regulated (FC < 0.2, FDR < 0.001) in 10 W-vs-20 W and examined their expression levels in three cultivars ‘Eshi 1′, ‘Youhou’, and ‘Mopanshi’. A total of 11 unigenes were selected for qRT-PCR analysis (Fig. 6B). The expression levels of the 11 genes (i.e. *Unigene39486_All, Unigene24632_All, Unigene1955_All, CL3714.Contig4_All, Unigene10342_All, Unigene30211_All, Unigene_13648_All, CL2365.Contig2_All, Unigene5625_All, CL11743.Contig1_All,* and *CL3961.Contig1_All*) that are annotated as *PAL, C4H, 4CL, CHS, CHI, F3H, F3*′*5*′*H, ANR, ANS, MATE*, and *LAC*, respectively. These genes encode enzymes of the PA biosynthesis pathway. The expression of most genes (*PAL, C4H, 4CL, CHS, CHI, F3H, F3*′*5 H*′, *ANR, ANS*, and *MATE*) was synchronously down-regulated from 2.5 WAB and was almost below the detection limit after 10 WAB in JPCNA and after 15 WAB in CPCNA (Fig. 6B). The relative expression level in non-PCNA was higher than that in CPCNA, followed by JPCNA. The expression of *LAC_CL3961.Contig1_All* was up-regulated in the non-PCNA type after 5 WAB (Fig. 6B), consistent with the function of *LAC* genes, which might participate in PA oligomerization/polymerization. Thus, the expression levels of these PA biosynthesis-related genes correspond to the tannin accumulation pattern of these three cultivars, in that the termination of tannin accumulation occurs earlier in JPCNA (10 WAB) and later in CPCNA (20 WAB). However, this accumulation sustainably increases in non-PCNA until fruit ripening (Fig. 1C).

### Proteomic analysis of differentially expressed proteins (DEPs) in persimmon fruits undergoing de-astringency naturally or with water treatment

**Figure 7:**
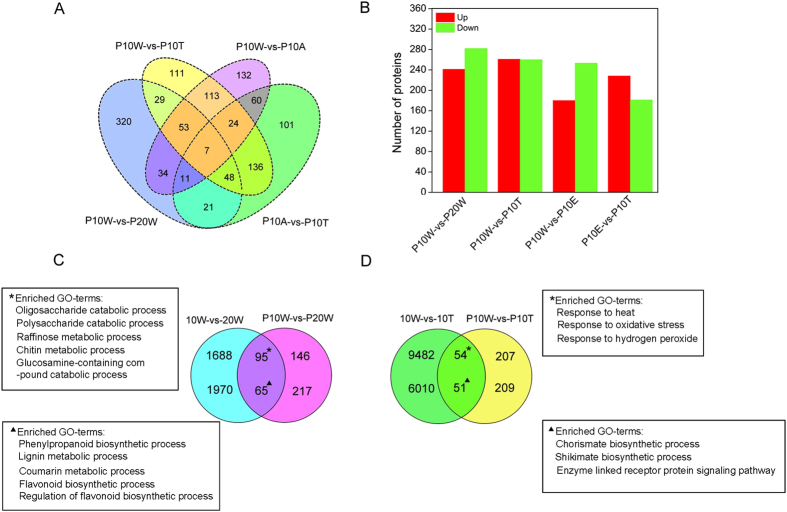
Overview of the differential expression proteins and enriched GO terms of shared up- and down-regulated unigenes and proteins in natural and water-treated de-astringency. (DEP with a *P* value of < 0.05 and an expression level change of at least 1.2-fold). (**A**) Venn diagram showing the number of differentially expressed proteins in fruit developmental stages and water treatment. (**B**) Number of proteins that are differentially expressed in 10 W-vs-20 W, 10 W -vs-10 T, 10 W-vs-10 A, and 10 A-vs-10 T. Red bars are up-regulated proteins and green bars are down-regulated proteins. Number of shared up-regulated (**C**) and down-regulated (**D**) unigenes and proteins with associated GO terms in natural and water-treated de-astringency. 10 W: fruits sampled at 10 weeks after bloom (WAB); 20 W: fruits sampled at 20 WAB; 10 T: fruits sampled at 10 WAB and then treated with 40 °C water for 12 h; 10 A: fruits sampled at 10 WAB and then treated with 25 °C air for 12 h. P10 W, P20 W, and P10T the samples 10 W, 20 W, and 10 T used for iTRAQ-based proteomic analysis, respectively.

To identify the differentially expressed proteins underlying natural and water-treated de-astringency, the sample used for RNA-seq (i.e. 10 W, 20 W, 10 T, and 10 A) were also used for the proteomic analysis. A total of 22,261 unique peptides were identified (Supplementary Dataset 12). These 22,261 peptides were matched to 4,954 unique protein groups in 8 samples (P10W, P20W, P10T, and P10A each with two biological replicates) (Supplementary Dataset 13). Multidimensional scaling (MDS) plot of 8 iTRAQ samples based on expression of all matched proteins were performed analysis the correlation between two iTRAQ datasets for each treatment (Supplementary Fig. 4). The two replicated for each treatment cluster together, except P10W. A total of 523 proteins were differentially expressed in natural de-astringency (P10 W-vs-P20 W), of which 241 proteins were up-regulated and 282 down-regulated (Fig. 7B and Supplementary Dataset S14). In addition, there were a total of 521 DEPs in artificial de-astringency (P10 W-vs-P10 T), of which 260 and 261 were up- and down-regulated, respectively (Fig. 7B and Supplementary Dataset S15). To quantify background protein expression changes in the persimmon transcriptiome, the 25 °C air-treated fruits at 12 h posttreatment (P10A) were also processed for protein profiling. We compared the P10 W vs P10 A (P10 W-vs-P10 A) and P10A vs P10T (P10 W-vs-P10 T), in order to minimize proteins that related to de-astringency. The up- and down-regulated genes in each pairwise comparison are shown in Venn diagram (Fig. 7A).

### The proteins involved in PA biosynthetic pathway were also down-regulated

With respect to the proteins involved in PAs biosynthesis, all of the proteins that were expressed in both natural and artificial de-astringency were investigated. By mapping proteins to PA biosynthesis pathways, a total of 50 proteins were found (Table 3), of which 20 proteins are differentially expressed in natural de-astringency, and only two proteins (i.e. one F3′H and one GST) slightly increased, 18 of them were down regulated. 13 proteins differentially expressed under water treatment (Table 3), except 6 GST-like protein were up-regulated, other proteins involved in PA biosynthetic pathway, namely, DAHPS, DHD, 4CL, F3′H, and F3′5′H were down-regulated. The expression pattern of proteins is highly consistent with the DEGs involved in PA biosynthetic pathway.

### Conjoint analysis of DEPs and DEGs in two de-astringency processes

The conjoint analysis of DEGs and DEPs was performed between the natural and artificial de-astringency processes. In natural de-astringency, 95 and 65 genes/proteins were shared up- and down-regulated, respectively (Fig. 7C), meanwhile, 54 and 51 genes/proteins were shared up- and down-regulated in water-treated de-astringency, respectively (Fig. 7D). Both up- and down-regulated genes/proteins were subjected to a GO enrichment analysis and semantic clustering. The 65 shared down-regulated genes/proteins were significantly enriched in the GO terms of phenylpropanoid biosynthetic process and flavonoid biosynthetic process (Fig. 7C), and these GO terms were mainly associated with PA biosynthesis. The 95 shared up-regulated genes/proteins were mainly enriched in sugar metabolism (i.e. oligosaccharide catabolic process, polysaccharide metabolic process, raffionse metabolic process, and glucosamine-containing compound catabolic process) (Fig. 7C). In de-astringency with water treatment, the GO term of 51 shared down-regulated genes/protein were related to primary chorismate biosynthesis process and shikimate biosynthesis were quite abundant (Fig. 7D). In addition, 54 shared up-regulated genes/proteins, the significantly enriched GO terms that were related to the response to heat and stimulus (Fig. 7D).

### Identification of de-astringency-specific transcription factor (TFs) during fruit astringency removal

**Figure 8:**
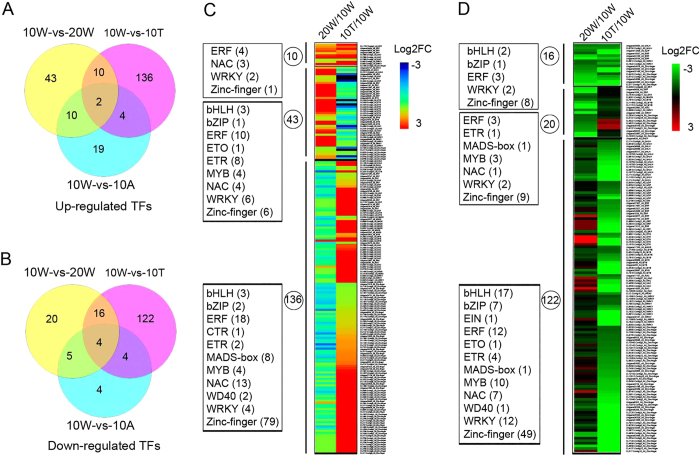
Overview of the differentially expressed TFs in natural and water-treated astringency removal. Venn diagram showing the number of up-regulated (**A**) and down-regulated (**B**) TFs that in natural (10 W-vs-20 W) and after water-treated (10 W-vs-10 T) de-astringency. The expression level and classification of TFs that unique up-regulated (**C**) and down-regulated (**D**) in 10 W-vs-20 W and in 10 W-vs-10 T, respectively. The intensity of the log2 fold change (−3 to 3) is indicated using rainbow scheme; red, up-regulated; blue (left heat map) or green (right heat map), down-regulated. 10 W: fruits sampled at 10 weeks after bloom (WAB); 20 W: fruits sampled at 20 WAB; 10 T: fruits sampled at 10 WAB and then treated with 40 °C water for 12 h; 10 A: fruits sampled at 10 WAB and then treated with 25 °C air for 12 h.

To identify TFs that related to persimmon fruits de-astringency, we carried out a targeted analysis of TFs that response to de-astringency, and minimized with genes differentially expressed under 25 °C air treatment, because in this condition fruits cannot remove its astringency. We found 43 and 136 TFs are especially up-regulated in 10 W-vs-20 W and 10 W-vs-10 T, respectively. And 10 TFs (i.e. 4 *ERF*, 3 *NAC*, 2 *WRKY*, and 1 *zinc finger transcription factor*) shared up-regulated in two de-astringency process (Fig. 8A). For down-regulated TFs, 20 and 122 are especially up-regulated in 10 W-vs-20 W and 10 W-vs-10 T, respectively. And 16 TFs (i.e. 2 *bHLH,* 1 *bZIP*, 3 *ERF*, 2 *WRKY*, and 8 *zinc finger transcription factor*) shared down-regulated in two de-astringency process (Fig. 8B).

## Discussion

Because persimmon being a perennial and hexaploid, limited information is available on the molecular mechanism underlying the fruit (de)astringency of CPCNA persimmon. Here, we reported a comprehensive transcriptome and proteome study to characterize the gene/protein expression profiles of natural and water-treated de-astringency in CPCNA fruit. We identified differentially expressed genes and proteins, and characterized the functional characteristics of DEGs and DEPs in two processes of astringency removal that could be further exploited to help understanding the molecular events during CPCNA natural astringency removal.

Previous reports demonstrated that soluble tannin concentration was markedly reduced in the PCNA type compared with the non-PCNA type at an early stage of fruit development^10^,^12^,^39^. In this study, we found the astringency removal in CPCNA is in the late stage of fruit development (i.e. after 20 WAB), which is far than in JPCNA (i.e. 10 WAB), however the soluble tannin in non-PCNA ‘Mopanshi’ maintain high level until full ripening (i.e. 25 WAB). The PA biosynthetic pathway was severely blocked in late stage of fruit development. Many of GO terms of down-regulated genes and proteins in natural de-astringency were related to the PA biosynthesis-related pathway (Fig. 3D and Supplementary Dataset S7). Expression of genes related to PA biosynthetic, names, *DHQS, DHD, SK, C4H, CHS, CHI, F3H, F3*′*H, F3*′*5*′*H, DFR, ANS*, and *ANR* showed highly reduced (Fig. 6A) and genes corresponding proteins were also down-regulated (Table 3). However, we found *Glutathione S-transferase-like (GST-like*) gene was up-regulated both in natural and water-treated de-astringency. In *Arabidopsis, TT19* encoding GST transporter is involved in the accumulation of both anthocyanins and proanthocyanidins^14^, however, the primary function of GSTs is generally considered to be the detoxification of both endogenous and xenobiotic compounds^40^,^41^,^42^. In a variety of plants, specific GSTs are reported to be induced upon infection, in response to treatment with heat shock, hydrogen peroxide, plant hormones, dehydration, wounding and senescence^40^. Thus, the up-regulated *GST-like* genes in this experiment might response to heat stress (i.e. 40 °C water treatment) or biotic and abiotic stress (i.e. plant hormones). Furthermore, we performed qRT-PCR to validate the expression of 11 key structural genes in the three cultivars (Fig. 6B). The expression of most of these genes, except for that of *LAC_CL3961.Contig1_All*, was simultaneously down-regulated from 2.5 WAB and was almost below the detection limit after 10 WAB in JPCNA and after 15 WAB in CPCNA and non-PCNA, but the average expression levels of these genes were approximately 2- and 11-fold higher in non-PCNA than in CPCNA and JPCNA at 10 WAB, respectively. The PCNA-type-specific down-regulated PA pathway genes coincided with the reduced PA amount in PCNA type (Fig. 1C). Expression of most of genes (i.e. *DkPAL, DkCHS, DkCHI, DkF3H, DkF3*′*5*′*H, DkDFR, DkANS*, and *DkANR*) involved in PA biosynthetic pathway was synchronously down-regulated from 5 WAB and was almost below the detection limit after 7 WAB in JPCNA^39^. And the expression of genes (*PAL, CHS, F3H, DFR, and ANR*) was continuous in the CPCNA, despite the termination of tannin cell development (i.e. 10 WAB)^4^. This results was consistent with the PA accumulation pattern in three types of persimmon (Fig. 1C), and also proved that the astringency removal naturally in CPCNA is different from JPCNA, the “dilution effect” for JPCNA natural de-astringency^34^ was not adequate to cause CPCNA persimmon fruits to loss its astringency. It is very interesting that decrease slope rate of the insoluble tannin concentration become reduced 10 to 25 WAB and have a slightly increase during 10 to 20 WAB, but the soluble tannin concentration continues decrease rapidly in CPCNA. This phenomenon is not observed in JPCNA and non-PCNA (Fig. 1C). Thus, we presumed that there may be a “coagulation effect” that soluble tannins converted to insoluble during late stage of CPCNA fruit development and caused de-astringency.

Acetaldehyde is the product of pyruvate produced by glycolysis. As mentioned previously, acetaldehyde is one of the main compounds that render the soluble tannin insoluble and cause de-astringency. Synthesis of acetaldehyde is generally catalysed by PDC, which converts pyruvate to acetaldehyde. ADH is then involved in the potentially reversible interconversion of acetaldehyde and ethanol^43^. In persimmon fruit, eight *DkADH* and *DkPDC* genes were isolated, and *DkADH1 and DkPDC2* have been suggested to be key genes involved in persimmon astringency removal^29^,^30^. Compared to 25 °C water treatment, 40 °C water showed more effective in inducing de-astringency, but the fruits in 25 and 40 °C air cannot lose their astringency (Fig. 1D). The soluble tannin was sharply decreased after 12 h treatment, company with insoluble tannin rapidly increased of fruits treated with 40 °C water (Fig. 1D). This results suggested that anaerobic condition caused fruits astringency removal, heat treatment only accelerate this process but was not necessary to remove fruits astringency. Both the KEGG pathways and GO enrichment results implied that glycolysis process and pyruvate biosynthesis is significantly enriched in underlying water-treated fruits (Fig. 3B,E). The genes for glycolysis process (i.e. *HXK, PGI, PFK, ALD, TPI, GAPDH, PGK, PGAM, NSE*, and *PK*), and specific for acetaldehyde biosynthetic (i.e. 14 *PDC-like* and 7 *ADH-like* genes) were markedly up-regulated (Fig. 5A). This is consistent with previous report that acetaldehyde was found to accumulate significantly in the flesh following the treatment of fruits with warm water^17^, and acetaldehyde being the main compound involved in the insolubilization of soluble tannin^22^,^23^,^24^.

Based on the GO enrichment analysis up- and down-regulated genes shared in natural and water-treated de-astringency. We found 228 shared up-regulated genes were significantly enriched in the GO terms related to primary sugar metabolism were quite abundant (Fig. 4B and Supplementary Dataset S5). Several genes (i.e. 2 *PFK*, 1 *ALD*, 1 *GAPDH*, and 1 *PK*) that involved in glycolysis were also up-regulated in natural astringency removal (Table 1). Phosphofructokinase (PFK) and Pyruvate Kinase (PK) are two key regulatory enzymes in plant glycolysis^44^. Salminen and Young^45^ found that PFK, an important regulatory enzyme in the glycolytic pathway, was activated during ripening of banana fruit. ALD is a ripening related enzymes in strawberry fruits^46^. In our pervious study, six *DkPK* genes were isolated from CPCNA ‘Eshi 1′, and *DkPK1* might play an important role in CPCNA natural de-astringency^31^. We also obtained three *PDC-like* unigenes (i.e. *CL5884.Contig2_All, PDC_unigene2205_All, and Unigene57828_All*) specific up-regulated in natural de-astringency process (Table 2 and Fig. 5A). Mo *et al*.^30^ transiently over-expressed *DkPDC2* in persimmon leaves, resulted in a significant decrease in the amount of soluble PAs. We performed qRT-PCR to measure the expression of three *PDC-like* genes in three cultivars (Fig. 5B) and found that all three of the *PDC-like* genes were up-regulated during the development of persimmon fruits. *PDC_CL5884.Contig2_All* was specifically up-regulated after 20 WAB compared to that in non-PCNA. However, there were no *ADH-like* genes up-regulated in natural de-astringency (Table 1). We examined one *ADH-like* gene *CL1015.Contig8*, which highly induced in water treatment (Fig. 5A). The qRT-PCR also showed that *ADH_CL1015.Contig8* was down-regulated during fruit development. In addition, the aldehyde dehydrogenase family 2 gene *(ALDH2*) was suggested to be a regulator of persimmon de-astringency under ethanol treatment and catalyses the conversion of acetaldehyde to acetic acid^35^. The expression of *ALDH2_Unigene17942_All* was significantly down-regulated after 10 WAB in CPCNA and non-PCNA. This decrease in *ALDH2* expression might resulted in acetaldehyde accumulation. Thus, these results indicated that *PDC_CL5884_All, PDC_Unigene2205_All* and *ALDH2_Unigene17942_All* might be involved in CPCNA natural de-astringency.

In persimmon fruits, two Myb-TFs (*DkMyb2* and *DkMyb4*) were suggested to be involved in PA biosynthesis^39^,^47^. And reduction in the *DkMyb4* expression causes the JPCNA specific down-regulation of PA biosynthesis at the early stage of fruit developmental and resultant non-astringent trait. *DkbZIP5* was found response to seasonal abscisic acid signal act as a *DkMYB4* regulator and modification of PA accumulation in JPCNA persimmon fruits^48^. However, a few transcription factor have been suggest to be involved in the de-astringency response in CPCNA persimmon fruit. Only six transcription factors were characterized can trans-activate soluble tannin coagulation related genes (i.e. *DkADH1, DkPDC2*, and *DkPDC3*), these include four *DkERF* genes (*DkERF9/10/19/22*), one *MYB* transcription factor (*DkMYB6*), and one *bZIP* gene (*DkTGA1*)^29^,^33^,^49^,^50^. Here, 43 TFs (i.e. 10 *ERF*, 8 *ETR*, 1 *ETO*, 6 *WRKY*, 6 *zinc finger*, 4 *NAC*, 4 *MYB*, 1 *bZIP*, and 3 *bHLH*) were specific up-regulated in natural de-astringency. And 10 TFs (i.e. 4 *ERF*, 3 *NAC*, 2 *WRKY*, and 1 *zinc finger*) shared up-regulated in two de-astringency process (Fig. 8A). Among these up-regulated TFs, we found ethylene-related TFs was enriched in natural de-astringency, followed by *WRKY* and *NAC* transcription factors. Furthermore, the many GO terms found for the up-regulated genes also related to ethylene response (Supplementary Dataset S5). This results implied that these TFs (i.e. ethylene-related and natural de-astringency specific expressed TFs) may involve in CPCNA natural astringency removal via activating glycolysis/acetaldehyde pathway genes expression and convert soluble tannin into insoluble by acetaldehyde and resultant non-astringent at later stage of fruit development.

**Figure 9:**
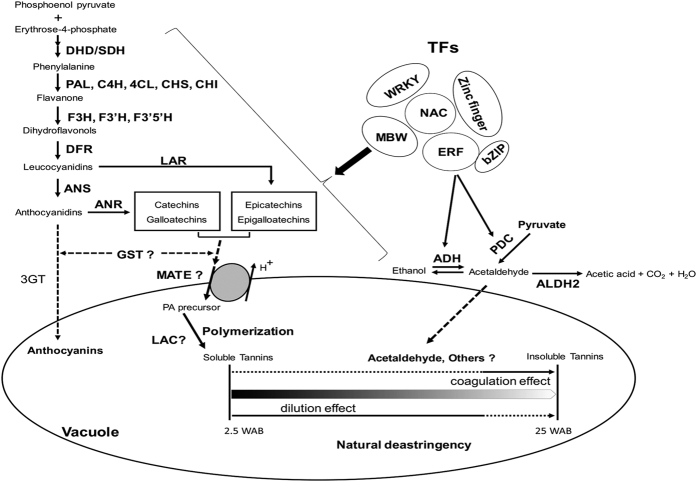
A hypothesis for PA biosynthesis and natural de-astringency of C PCNA fruits. DAHPS, 3-deoxy-d-arabino-heptulosonate 7-phosphate synthase; DHD/SDH, 3-dehydroquinate dehydratase/shikimate 5-dehydrogenase; PAL, phenylalanine ammonia-lyase; C4H, cinnamate-4-hydroxylase; 4CL; 4-coumarate: coenzyme A ligase; CHS, chalcone synthase; CHI, chalcone isomerase; F3H, flavanone-3-hydroxylase; F3′H, flavonoid 3′-hydroxylase; F3′5′H, flavonoid 3′5′-hydroxylase; DFR, dihydroflavonol 4 reductase; ANS, anthocyanidin synthase; LAR, leucoanthocyanidin reductase; ANR, anthocyanidin reductase; 3GT, 3-glycosyltransferase; GST, glutathione S-transferase; MATE transporter, multi-drug and toxic compound extrusion transporter; LAC, laccase; ADH, alcohol dehydrogenase; PDC, pyruvate decarboxylase; ALDH2, acetaldehyde dehydrogenase 2; MBW, MYB-bHLH-WDR; bZIP, basic region-leucine zipper; ERF, ethylene responsive factor; NAC, NAM-ATAF1,2,-CUC2.

Based on our data and previous studies, we propose a hypothesis for the natural de-astringency of CPCNA fruit (Fig. 9). The biosynthesis of PA precursors is believed to occur on the cytosolic face of the endoplasmic reticulum surface; these precursors will first be transported into the vacuole by GST and MATE transporters. LAC has been suggested to participate in PA oligomerization/polymerization^16^,^51^. In CPCNA fruits, PA synthesis was until at late stage of fruit development (20 WAB), the “dilution effect” was not enough to cause CPCNA fruits to loss its astringency, the excess soluble tannin may insolubilized with acetaldehyde continued to fully ripen and resultant non-astringent.

In summary, an integrated analysis based on transcriptome and proteome were performed in CPCNA de-astringency. The differentially expressed genes and proteins undergoing de-astringency processes were identified. Based on the GO and pathway enrichment analysis, we found water treatment strongly up-regulated glycolysis/acetaldehyde metabolism, which confirm previous study that acetaldehyde was the main components for water-treated de-astringency. In natural de-astringency process genes/proteins involved in PA biosynthesis was markedly reduced and the significant GO terms found for the set of up-regulated genes were related to sugar metabolism and ethylene response pathway. The acetaldehyde biosynthesis-related genes and TFs that specific expressed in natural de-astringency were identified. These soluble tannin coagulation-related genes may help us understanding the molecular event of CPCNA natural de-astringency and persimmon breeding in future.

## Methods

### Plant materials

The persimmon (*Diospyros kaki* Thunb.) fruits of ‘Eshi 1′ (CPCNA type), ‘Youhou’ (JPCNA type) and ‘Mopanshi’ (non-PCNA type) were obtained from the Persimmon Repository of Huazhong Agricultural University, Wuhan, China. Three biological replicates were collected from three individuals, and each biological replicates contained 10–15 fruits. These samples were collected 2.5, 5, 10, 15, 20, and 25 weeks after bloom (WAB). Another group of ‘Eshi 1′ fruits sampled at 10 WAB (three biological replicates) were then treated with water (25 and 40 °C) and their corresponding control (25 and 40 °C air treatment) were collected at 12 h, 24 h, 36 h, and 48 h after treatment. All of the fruits were peeled, and the flesh at the equator was collected, immediately frozen in liquid nitrogen and maintained at −80 °C until use. The fruits sampled at 10 WAB (10 W), 20 WAB (20 W), water-treated (40 °C·12 h) (10 T), and air-treatment (25 °C·12 h) (10 A) were select for RNA-seq. The RNA-seq corresponding samples (i.e. P10 W, P20 W, P10T, and P10 A) were also used for iTRAQ-based proteomic analysis.

### Analysis of the soluble and insoluble tannin contents

The concentrations of the soluble and insoluble tannins in the samples were measured by the Folin-Ciocalteu method^36^. Soluble tannins were also examined by the printing method^52^, which is a convenient way to measure the soluble tannin content in fruit. Due to reaction between FeCl_2_ and soluble tannins, the darker is the resulting product, the higher is the soluble tannin content in the fruits.

### RNA isolation, cDNA library construction and sequencing

The total RNA was extracted using TRIzol Reagent (Invitrogen, USA) according to the manufacturer’s protocol. A NanoDrop 2000 spectrophotometer (Thermo Scientific, USA) and gel electrophoresis were used determine the quality and integrity of the total RNA. For each sample, the RNA was extracted from three fruit flesh biological replicates, mixed in equal quantities and stored at −80  °C before sequencing. A total of 5 μg of total RNA for each tissue sample with RNA integrity number (RIN) values greater than 8 was used for library construction. Briefly, mRNA was purified from total RNA using poly-T oligo-attached magnetic beads. Following purification, the mRNA was fragmented into small pieces using divalent cations under elevated temperature. Then, the cleaved RNA fragments were reverse-transcribed to create the final cDNA library in accordance with the protocol for the mRNA-Seq sample preparation kit (Illuminar, San Diego, USA). Then, three cDNA libraries were sequenced on an Illumina HiSeq™ 2000 platform, and reads were generated in 100-bp paired-end format.

### *De novo* transcriptome assembly

For all libraries, raw sequencing reads of low quality (Phred quality score <20 and bases from the 3′ ends of reads) were filtered out, and adapter/primer contaminants and duplicated reads were also deleted, after which clean reads of high quality were obtained. All of the downstream analyses were based on these clean data of high quality. Reads from all 12 samples were concatenated and a reference assembly created using the Trinity software package (version r2013-02-25)^53^ with min_kmer_cov and was set to 4 and all other parameters set to default. The assembled sequences were called unigenes, and the TGICL software package^54^ was used to remove spliced and redundant sequences to acquire non-redundant unigenes that were as long as possible. The clustered unigenes shared more than 70% sequence similarity and were named using the prefix CL, and single unigenes were named using the prefix Unigene.

### Functional annotation of the transcriptome

The unigenes from the final transcriptome were aligned with sequences in the NR (NCBI non-redundant protein sequences), Swiss-Prot (a manually annotated and reviewed protein sequence database), KEGG (Kyoto Encyclopaedia of Genes and Genomes), and COG (Cluster of Orthologous Groups of proteins) protein databases using BLASTX with an e-value threshold of 1e-5. The directions of the unigene sequences were determined based on the best alignment results over all of these databases. When conflicting results were produced from different databases, a priority order of NR, Swiss-Prot, KEGG, and COG was followed. ESTScan software^55^ was used to determine the coding regions and sequence orientation, when a unigene could not be aligned to any of the databases.

### Differential expression analysis

The gene expression levels were estimated by RSEM^56^ for each sample. Clean data were mapped back onto the assembled transcriptome and then the read count for each gene was obtained from the mapping results. The differential expression analysis was carried out using the Bioconductor package EdgeR^57^. The differentially expressed genes (DEGs) were selected by a cutoff for pairwise comparisons with a fold change greater than 2 (false discovery rate (FDR) *P* value ≤ 0.001).

### Protein extraction

Fruits were ground in liquid nitrogen with 0.1 mg of polyvinylpyrrolidone (PVP) and precipitated by 10% (v/v) trichloroacetic acid (TCA) in acetone at −20 °C. Then, the samples were incubated at −20 °C for 2 h after thorough mixing. The proteins were collected by centrifugation at 30,000 g at 4 °C for 30 min. The protein pellets were washed three times with cold acetone to remove TCA. Each protein pellet was air dried, resuspended in 30-times volume of SDT buffer (4% SDS, 100 mM Tris-HCl, 1 mM DTT, pH 7.6), mixed and boiled for 5 min. The suspension was sonicated and then boiled for 15 min. After centrifugation at 4 °C at 14,000 g for 15 min, the supernatant was filtered through 0.22-μm filters and quantified using the BCA Protein Assay Kit (Bio-Rad, USA).

### iTRAQ labelling and SCX fractionation

Protein digestion was performed according to the FASP procedure described by Wisniewski, *et al*.^58^ and the resulting peptide mixture was labelled using the 8-plex iTRAQ reagent according to the manufacturer’s instructions (Applied Biosystems). Peptides from fruits samples P10 W1, P10 W2, P20 W1, P20 W2, P10 T1, and P10 T2 were labelled with the iTRAQ tags 113, 114, 115, 116, 119, and 121, respectively. iTRAQ-labelled peptides were fractionated by SCX chromatography using the AKTA Purifier system (GE Healthcare). The dried peptide mixture was reconstituted and acidified with 2 ml of buffer A (10 mM KH_2_PO_4_ in 25% ACN, pH 2.7) and loaded onto a PolySULFOETHYL 4.6 × 100 mm column (5 μm, 200 Å, PolyLC Inc, Maryland, USA). The peptides were eluted at a flow rate of 1 ml/min with a gradient of 0–10% buffer B (500 mM KCl, 10 mM KH2PO4 in 25% of ACN, pH 2.7) for 2 min, 10–20% buffer B for 25 min, 20–45% buffer B for 5 min, and 50–100% buffer B for 5 min. The elution was monitored by absorbance at 214 nm, and fractions were collected every 1 min. The collected fractions (approximately 30 fractions) were finally combined into 6 pools and desalted on C18 Cartridges (Empore™ SPE Cartridges C18 (standard density), bed I.D. 7 mm, volume 3 ml, Sigma). Each fraction was concentrated by vacuum centrifugation and reconstituted in 40 μl of 0.1% (v/v) trifluoroacetic acid. All of the samples were stored at −80 °C until LC-MS/MS analysis.

### LC-MS/MS analysis based on Q EXACTIVE

The experiments were performed on a Q Exactive mass spectrometer that was coupled to Easy nLC (Thermo Fisher Scientific). An aliquot of 10 μl of each fraction was injected for nanoLC-MS/MS analysis. The peptide mixture (5 μg) was loaded onto a C18-reversed phase column (Thermo Scientific Easy Column, 10 cm long, 75-μm inner diameter, 3-μm resin) in buffer A (0.1% Formic acid) and separated with a linear gradient of buffer B (80% acetonitrile and 0.1% Formic acid) at a flow rate of 250 nl/min controlled by IntelliFlow technology over 140 min. MS data were acquired using a data-dependent Top 10 method to dynamically choose the most abundant precursor ions from the survey scan (300–1800 m/z) for HCD fragmentation. Determination of the target value is based on predictive Automatic Gain Control (pAGC). The dynamic exclusion duration was 60 s. Survey scans were acquired at a resolution of 70,000 at m/z 200, and resolution for HCD spectra was set to 17,500 at m/z 200. The normalized collision energy was 30 eV, and the underfill ratio, which specifies the minimum percentage of the target value likely to be reached at the maximum fill time, was defined as 0.1%.

### Protein identification and quantification

MS/MS spectra were searched using MASCOT (Matrix Science, London, UK; version 2.2) embedded in Proteome Discoverer 1.3 (Thermo Electron, San Jose, CA.) against a deduced protein database from RNA-Seq. We set “*P* value ≤ 0.05, |log2FC| ≥ 1.2” as the differentially expressed proteins (DAPs).

### GO and pathway enrichment analysis

Statistically significant enrichment of Gene Ontology (GO) terms and KEGG pathway was analysed using Blast2GO^59^ and KAAS (http://www.genome.jp/tools/kaas), respectively. The enrichment analysis was performed using custom Perl scripts. These analyses were performed for all annotated differentially expressed genes/proteins in comparisons of natural and water-treated de-astringency, and for the up- and down-regulated genes separately. The background used for the GO enrichment analysis comprised all of the annotated persimmon transcripts. The outcome of the GO enrichment analysis was used for semantic clustering using REVIGO (http://revigo.irb.hr/) with default settings in order to identify non-redundant sub-sets of GO terms^60^.

### Quantitative real-time PCR validation of differentially expressed genes (DEGs) and proteins (DEPs)

qRT-PCR was performed to validate gene expression. cDNA was synthesized from 1.0 μg of RNA using the PrimeScript RT Kit with gDNA Eraser (TaKaRa, Dalian, China) according to the manufacturer’s protocol. qRT-PCR was performed with a real-time PCR instrument (QuantStudio 7 Flex Real-Time PCR system, Applied Biosystems) using SYBR^®^ Premix Ex TaqTM II (TaKaRa). *DkActin* (accession no. AB473616) was used as an internal reference, each sample was analysed in quadruplicate, and all of the primers are listed in Supplementary Table S1.

## Additional Information

**How to cite this article:** Chen, W. *et al*. An integrated analysis based on transcriptome and proteome reveals deastringency-related genes in CPCNA persimmon. *Sci. Rep.* **7**, 44671; doi: 10.1038/srep44671 (2017).

**Publisher's note:** Springer Nature remains neutral with regard to jurisdictional claims in published maps and institutional affiliations.

## Supplementary Material

Supplementary Information

Supplementary Dataset S1

Supplementary Dataset S2

Supplementary Dataset S3

Supplementary Dataset S4

Supplementary Dataset S5

Supplementary Dataset S6

Supplementary Dataset S7

Supplementary Dataset S8

Supplementary Dataset S9

Supplementary Dataset S10

Supplementary Dataset S11

Supplementary Dataset S12

Supplementary Dataset S13

Supplementary Dataset S14

Supplementary Dataset S15

## Figures and Tables

**Table 1:** Acetaldehyde metabolism-related genes expression undergoing natural and water-treated de-astringency.

Gene	Enzyme	All^a^	10W-vs-20W^b^		10W-vs-10T^c^		10W-vs-10A^d^	
			Up	Down	Up	Down	Up	Down
*HXK*	Hexokinase	15	0	0	3	0	0	1
*PGI*	Phosphoglucose Isomerase	40	0	0	3	0	0	0
*PFK*	Phosphofructokinase	23	2	0	4	1	0	1
*ALD*	Aldolase	28	1	0	3	2	0	0
*TPI*	Triphosphate isomerase	4	0	0	1	0	0	0
*GAPDH*	Glyceraldehyde-3-phosphate Dehydrogenase	47	1	1	5	2	0	2
*PGK*	Phosphoglycerate Kinase	20	0	0	2	0	0	0
*PGAM*	Phosphoglycerate Mutase	26	0	0	6	2	1	0
*NSE*	Enolase	11	0	0	3	0	0	0
*PK*	Pyruvate Kinase	32	1	0	5	3	0	1
*PDC*	Pyruvate decarboxylase	12	3	0	7	0	0	0
*ADH*	Alcohol dehydrogenase	75	0	8	14	2	1	0
*ALDH2*	Acetaldehyde dehydrogenase 2	3	1	0	1	0	0	0

^a^All, the total number of analysed uni-transcripts. ^b^10W-vs-20W, the ratio of genes expressed in ‘Eshi 1′ fruit at 20 WAB compared to those at 10 WAB. ^c^10W-vs-10T, the ratio of genes expressed in ‘Eshi 1′ fruit samled at 10 WAB and then treated with 40 °C water for 12 h compared to those in untreated fruits. ^d^10W-vs-10A, the ratio of genes expressed in ‘Eshi 1′ fruit sampled at 10 WAB and then treated with 25 °C water for 12 h compared to those in untreated fruits.

**Table 2:** PA biosynthesis-related genes expression undergoing natural and water-treated de-astringency.

Gene	Enzyme	All^a^	10W-vs-20W^b^		10W-vs-10T^c^		10W-vs-10A^d^	
			Up	Down	Up	Down	Up	Down
*DAHPS*	3-deoxy-d-arabino-heptulosonate-7-phosphate synthase	4	0	0	1	3	0	3
*DHQS*	3-dehydroquinate synthase	2	0	1	0	2	0	1
*DHD*	3-dehydroquinate dehydratase	16	0	1	0	0	0	1
*SK*	Shikimate kinase	15	0	2	2	0	0	0
*EPSPS*	5-enolpyruvylshikimate 3-phosphte synthase	1	0	0	0	1	0	0
*CS*	Chorismate synthase	5	0	2	1	0	0	0
*PAL*	Phenylalanine ammonia-lyase	14	0	0	10	13	0	5
*C4H*	cinnamate-4-hydroxylase	16	0	7	0	9	0	10
*4CL*	4-coumarate: coenzyme A ligase	23	2	0	1	6	0	4
*CHS*	Chalcone synthase	4	0	3	0	2	0	2
*CHI*	Chalcone isomerase	4	0	2	1	2	0	3
*F3H*	Flavanone 3-hydroxylase	8	0	1	1	3	0	1
*F3′H*	Flavonoid 3′-hydroxylase	6	0	3	0	3	0	3
*F3'5*′*H*	Flavonoid 3′5′-hydroxylase	5	0	1	0	2	0	1
*DFR*	Dihydroflavonol 4-reductase	41	2	1	4	1	2	1
*ANS*	Anthocyanidin synthase	1	0	1	0	1	0	1
*LAR*	Leucoanthocyanidin reductase	1	1	0	0	0	0	0
*ANR*	Anthocyanidin reductase	13	0	4	3	3	0	4
*GST*	Glutathione S-transferase	85	3	0	20	2	0	0
*MATE*	Multi-drug and toxic compound extrusion transporter	44	4	2	1	6	0	5
*LAC*	Laccase	13	1	2	4	4	0	2

^a^All, the total number of analysed uni-transcripts. ^b^10W-vs-20W, the ratio of genes expressed in ‘Eshi 1′ fruit at 20 WAB compared to those at 10 WAB. ^c^10W-vs-10T, the ratio of genes expressed in ‘Eshi 1′ fruit sampled at 10 WAB and then treated with 40 °C water for 36 h compared to those in untreated fruits. ^d^10W-vs-10A, the ratio of genes expressed in ‘Eshi 1′ fruit sampled at 10 WAB and then treated with 25 °C water for 12 h compared to those in untreated fruits.

**Table 3:** PA biosynthesis-related proteins expression undergoing natural and water-treated de-astringency.

Protein	Name	All^a^	P10W-vs-P20W^b^		P10W-vs-P10T^c^	
			Up	Down	Up	Down
DAHPS	3-deoxy-d-arabino-heptulosonate-7-phosphate synthase	3	0	1	0	2
DHQS	3-dehydroquinate synthase	1	0	1	0	0
DHD	3-dehydroquinate dehydratase	4	0	3	0	1
SK	Shikimate kinase	0	0	0	0	0
EPSPS	5-enolpyruvylshikimate 3-phosphte synthase	1	0	0	0	0
CS	Chorismate synthase	1	0	0	0	0
PAL	Phenylalanine ammonia-lyase	3	0	1	0	0
C4H	Cinnamate-4-hydroxylase	2	0	0	0	0
4CL	4-coumarate: coenzyme A ligase	3	0	2	0	1
CHS	Chalcone synthase	2	0	0	1	0
CHI	Chalcone isomerase	3	0	1	0	0
F3H	Flavanone 3-hydroxylase	2	0	2	0	0
F3′H	Flavonoid 3′-hydroxylase	2	1	1	0	1
F3'5′H	Flavonoid 3′5′-hydroxylase	1	0	1	0	1
DFR	Dihydroflavonol 4-reductase	4	0	0	0	0
ANS	Anthocyanidin synthase	1	0	1	0	0
LAR	Leucoanthocyanidin reductase	0	0	0	0	0
ANR	Anthocyanidin reductase	1	0	1	0	0
GST	Glutathione S-transferase	15	1	2	6	0
MATE	Multi-drug and toxic compound extrusion transporter	1	0	1	0	0
LAC	L-ascorbate oxidase	0	0	0	0	0
ADH	Alcohol dehydrogenase	12	0	1	0	0
PDC	Pyruvate decarboxylase	7	0	0	0	0
ALDH2	Acetaldehyde dehydrogenase 2	3	1	0	0	0

^a^All, the total number of analysed proteins. ^b^P10W-vs-P20W, the ratio of proteins expressed in ‘Eshi 1′ fruit at 20 WAB compared to those at 10 WAB. ^c^P10W-vs-P10T, the ratio of proteins expressed in ‘Eshi 1′ fruit sampled at 10 WAB and then treated with 40 °C water for 12 h compared to those in untreated fruits.
