# Supplementary material for: An integrated analysis based on transcriptome and proteome reveals deastringency-related genes in CPCNA persimmon
Source: Sci Rep. 2017 Mar 17;7:44671. doi: 10.1038/srep44671 (PMC5356345; doi:10.1038/srep44671)
Supplement: Supplementary Information [file srep44671-s1.docx]

Supplementary Information

**An integrated analysis based on transcriptome and proteome reveals deastringency-related genes in CPCNA persimmon**

Wenxing Chen^1^, Yalou Xiong^1^, Liqing Xu^1,^*, Qinglin Zhang^1^, Zhengrong Luo^1,2,^*

^1^Key Laboratory of Horticultural Plant Biology, Huazhong Agricultural University, Wuhan 430070, Hubei, China

^2^Hubei Collaborative Innovation Center for the Characteristic Resources Exploitation of Dabie Mountains, Huanggang Normal University, Huanggang 438000, Hubei, China

* Corresponding author

E-mail: luozhr@mail.hzau.edu.cn; liqingxu@mail.hzau.edu.cn


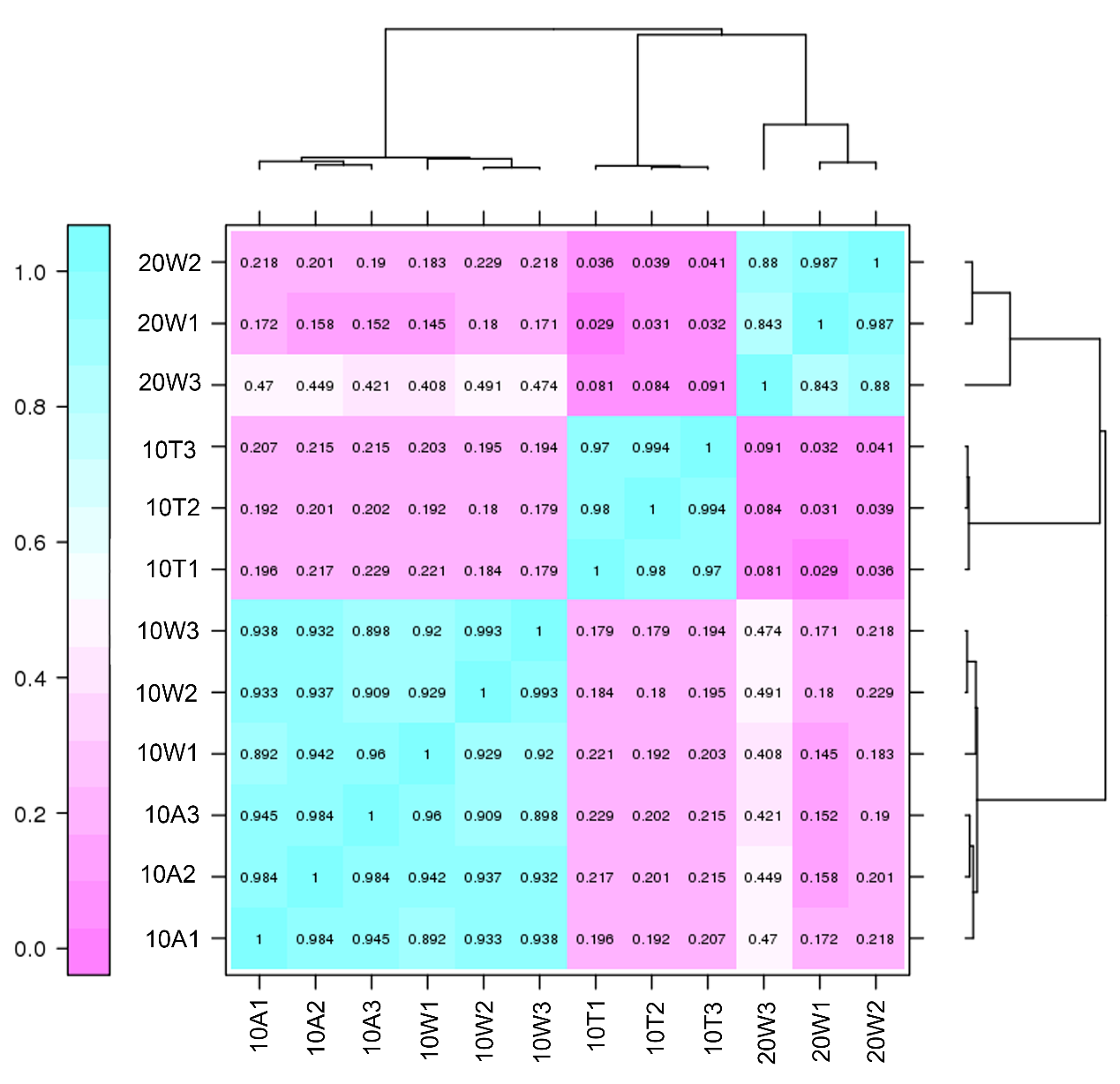


**Supplementary Figure S1.** Correlation matrix based on Euclidean distances and the complete linkage clustering methoed between the different samples showing that the three biological replicates cluster together. 10W: fruits sampled at 10 weeks after bloom (WAB); 20W: fruits sampled at 20 WAB; 10T: fruits sampled at 10 WAB and then treated with 40 °C water for 12 h; 10A: fruits sampled at 10 WAB and then treated with 25 °C air for 12 h.


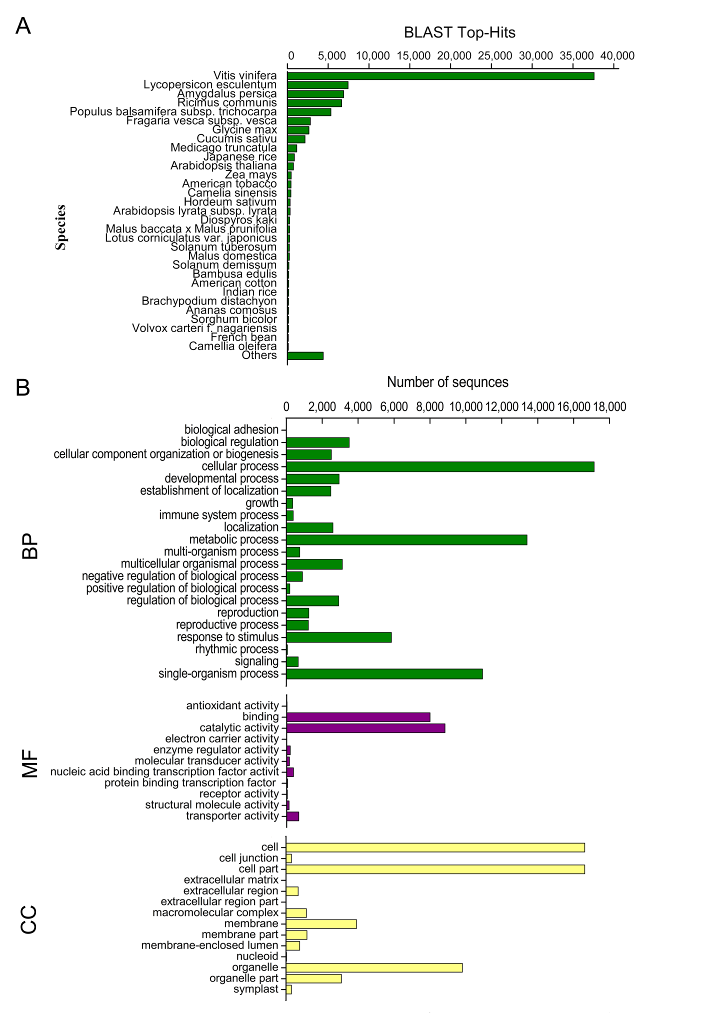


**Supplementary Figure S2. Blast2GO annotation of ‘Eshi 1’ persimmon transcriptome.** (**A**) Top-hit species distribution of the ‘Eshi 1’ fruit transcriptome showing the abundance of top hits to the sequences from members of the family Vitaceae. (**B**) GO category distribution of ‘Eshi 1’ persimmon transcripts among level 1 GO categories: biological process (BP), molecular function (MF), and cellular component (CC).


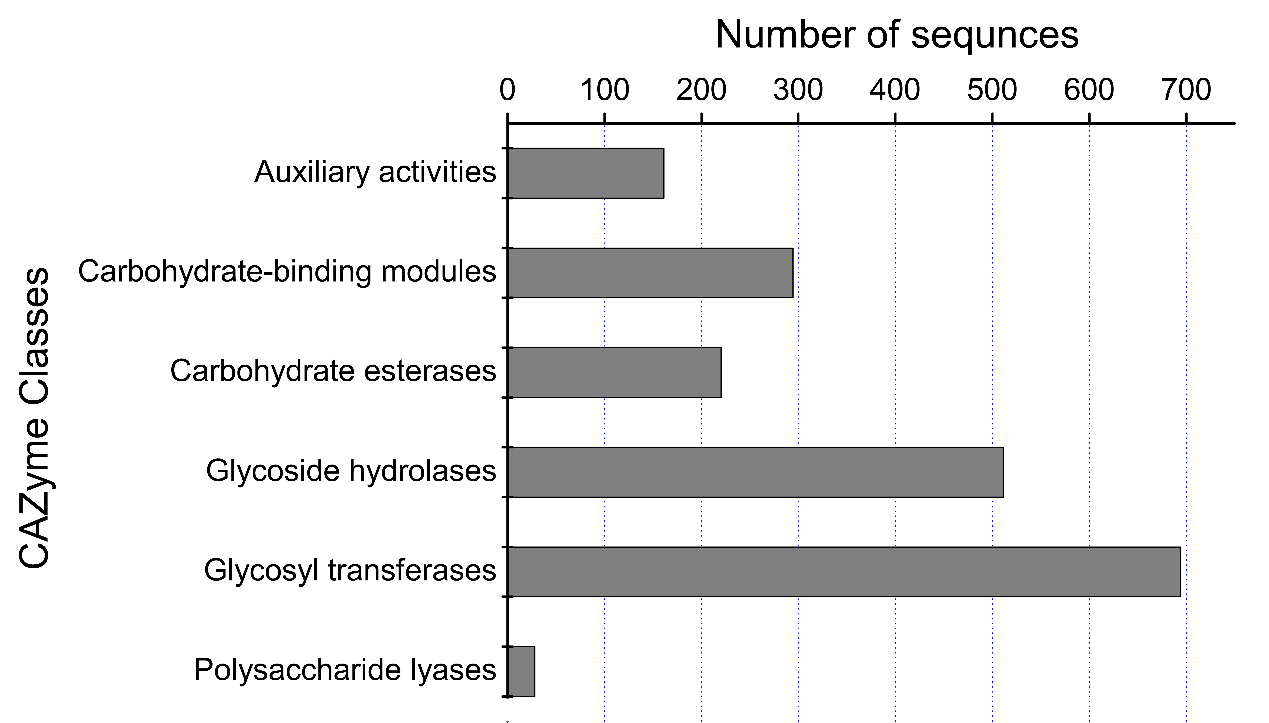


**Supplementary Figure S3.** Distribution of transcripts annotated as enzymes among different enzyme classes.


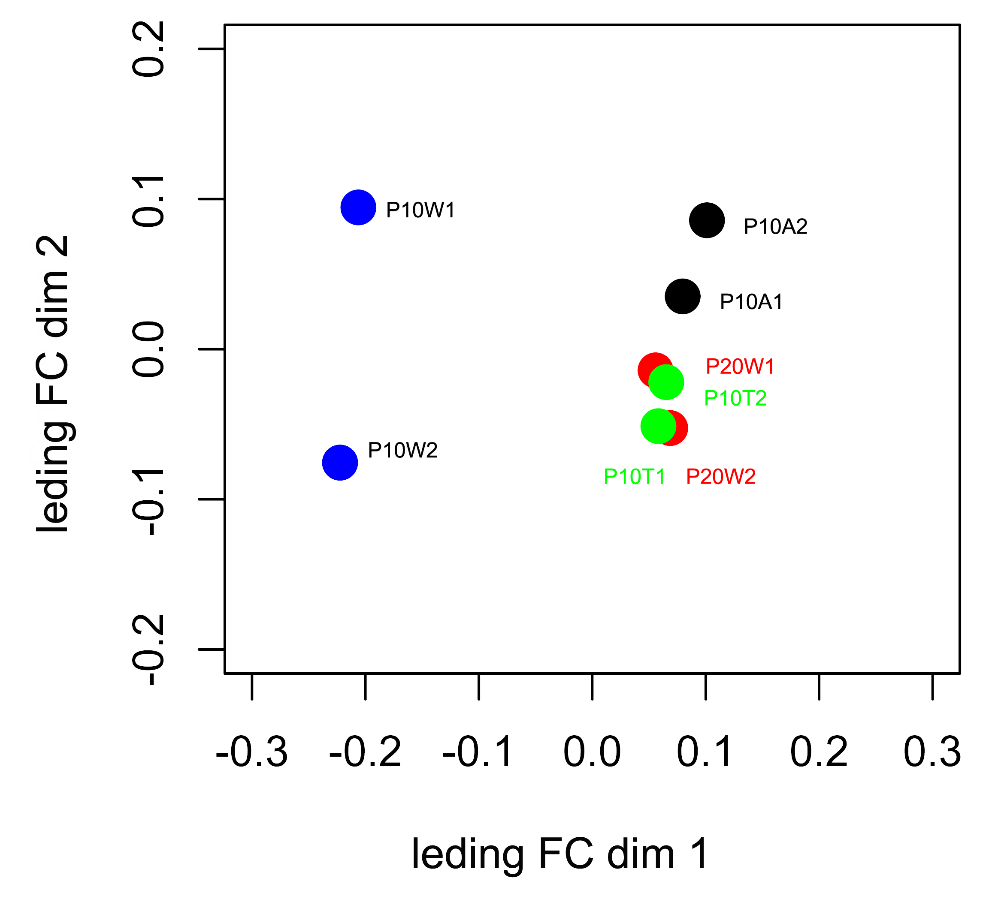


**Supplementary Figure S4.** Multidimensional scaling (MDS) plot of 8 iTRAQ samples based on expression of all matched proteins were performed analysis the correlation between two iTRAQ datasets for each treatment. The two replicated for each treatment cluster together, with the exception of P10W. P10W: fruits sampled at 10 weeks after bloom (WAB); P20W: fruits sampled at 20 WAB; P10T: fruits sampled at 10 WAB and then treated with 40 °C water for 12 h; P10A: fruits sampled at 10 WAB and then treated with 25 °C air for 12 h.

**Supplementary table S1.** Summary of the sequencing generated on the Illumina.

| Samples | Raw reads | Clean reads | Clean bases | GC Content | % ≥ Q30 |
| --- | --- | --- | --- | --- | --- |
| 10W_1 | 26,163,187 | 26,076,849 | 7,706,878,156 | 48.60% | 95.43% |
| 10W_2 | 30,505,959 | 30,402,239 | 8,999,372,878 | 47.81% | 95.19% |
| 10W_3 | 29,191,720 | 29,039,923 | 8,578,929,374 | 47.97% | 95.48% |
| 20W_1 | 33,194,922 | 33,078,740 | 9,817,830,408 | 47.97% | 95.33% |
| 20W_2 | 30,150,204 | 30,023,573 | 8,874,979,510 | 48.75% | 95.38% |
| 20W_3 | 25,472,237 | 25,395,820 | 7,504,828,892 | 47.93% | 95.46% |
| 10T_1 | 32,346,837 | 32,181,868 | 9,519,332,330 | 49.26% | 95.44% |
| 10T_2 | 27,676,202 | 27,416,046 | 8,079,718,068 | 49.12% | 95.13% |
| 10T_3 | 25,295,166 | 25,140,865 | 7,456,784,694 | 49.41% | 94.98% |
| 10A_1 | 26,023,264 | 25,926,978 | 7,671,634,808 | 47.54% | 95.24% |
| 10A_2 | 30,794,307 | 30,658,812 | 9,062,046,122 | 47.61% | 95.45% |
| 10A_3 | 36,005,007 | 35,839,384 | 10,585,685,038 | 48.35% | 95.30% |

Abbreviations: 10W: fruits sampled at 10 weeks after bloom (WAB); 20W: fruits sampled at 20 WAB; 10T: fruits sampled at 10 WAB and then treated with 40 °C water for 12 h; 10A: fruits sampled at 10 WAB and then treated with 25 °C air for 12 h.

**Supplementary table S2.** Summary of the C-PCNA persimmon assembly and unigenes annotation statistics.

| Total Unigenes | Mean length (nt) | N50 | NR | NT | Swiss-Prot | KEGG | COG | GO | Total %  annotated |
| --- | --- | --- | --- | --- | --- | --- | --- | --- | --- |
| 135,999 | 1,227 | 2,071 | 83,390 | 74,537 | 52,765 | 50,920 | 35,137 | 64,866 | 63.8 |

**Supplementary table S3.** Details of primers utilized for quantitative real time PCR.

| **Primer ID** | **Primers sequence** | **Gene description** |
| --- | --- | --- |
| *Unigene39486_All_F*  *Unigene39486_All_R* | 5’ CGAGCAGCACAACCAAGA 3’  5’ CAAATGCCTCAAATCAATAGC 3’ | *PAL* |
| *Unigene24632_All_F*  *Unigene24632_All_R* | 5’ TGAAGCGGAACCCAGAGG 3’  5’ CCTTGAGGCTTACGAACAGG 3’ | *C4H* |
| *Unigene1955_All_F*  *Unigene1955_All_R* | 5’ TCATCATCACCCAGTCCCAGTA 3’  5’ ATCGGTTTCGTTCGCTCC 3’ | *4CL* |
| *CL3714.Contig4_All_F*  *CL3714.Contig4_All_R* | 5’ ACCAACAGCGACCACAAGACC 3’  5’ CGCCATGTAGGCGCAGATA 3’ | *CHS* |
| *Unigene10342_All_F*  *Unigene10342_All_R* | 5’ TCCCTCCTGGTGCCTCTA 3’  5’ GCCTCCGATAACTGTTTGTT 3’ | *CHI* |
| *Unigene30211_All_F*  *Unigene30211_All_R* | 5’ ACCTACTTTTCATACCCGACCC 3’  5’ CCAGGCCCATCAACTTATCACT 3’ | *F3H* |
| *Unigene13648_All_F*  *Unigene13648_All_R* | 5’ CCGAGCATCCTCAAACGA 3’  5’ GGAATGTCTGACTCCTCCAAT 3’ | *F3'5'H* |
| *CL2365.Contig2_All_F*  *CL2365.Contig2_All_R* | 5’ AATGTTCCCACTGACTTCG 3’  5’ ACTCCACAGATTGGTCGTAG 3’ | *ANR* |
| *Unigene5625_All_F*  *Unigene5625_All_R* | 5’ TTCCGTGCTTTCACTTGG 3’  5’ TCTGGCTGAGGGCATTTT 3’ | *ANS* |
| *CL11743.Contig1_All_F*  *CL11743.Contig1_All_R* | 5’ AGCATAGCGGACGAAGGG 3’  5’ TGAGAATGTGGAGGAGAAAGAC 3’ | *MATE* |
| *CL7863.Contig5_All_F*  *CL7863.Contig5_All_R* | 5’ GGAAGCAGCTACACTTACAGA 3’  5’ ATTATTAGGGCACCATGGAC 3’ | *LAC* |
| *CL5884.Contig2-All_F*  *CL5884.Contig2_All_R* | 5’ CGTAAGAATGGCGGATGG 3’  5’ CGTTATGGAAGGCGATGAA 3’ | *PDC* |
| *Unigene2205-All_F*  *Unigene2205-All_R* | 5’ TGCCCTCGCTGAATCCTT 3’  5’ CCTCCCGCTTTCTGAACC 3’ | *PDC* |
| *Unigene57828_All_F*  *Unigene57828-All_R* | 5’ GTGAAGGCAACTGCTGGACAA 3’  5’ TTCGGAACGCACCTGAAATAC 3’ | *PDC* |
| *CL1015.Contig8-All_F*  *CL1015.Contig8_All_R* | 5’ ACGAAGGGAAACTGGAGA 3’  5’ GTAGGCAAGGATAGGTGGT 3’ | *ADH* |
| *Unigene17942_All_F*  *Unigene17942_All_R* | 5’ TAAGAGGGCAAATACCACC 3’  5’ ATCGCAGCATCAAAGACA 3’ | *ALDH2* |
| *Actin_F*  *Actin_R* | 5’ GAAGCACTGGGTGCTCTTCTG 3’  5’ CATGGAGAAAATCTGGCATCATAC 3’ | *DkActin* |
